# Resilience in people with epilepsy in Jordan: an interplay between demographics, clinical variables, and insomnia? A cross-sectional study

**DOI:** 10.1097/MD.0000000000046563

**Published:** 2025-12-12

**Authors:** Omar Gammoh, Mariam Al-Ameri, Alaa A. A. Al-Jabali, Hanan Abu-Alshaikh, Wail Ennab, Nada A. AlSaleh, Alaa A. AlSharif, Hayam A. AlRasheed, Sireen Abdul-Rahim Shilbayeh

**Affiliations:** a Department of Clinical Pharmacy and Pharmacy Practice, Faculty of Pharmacy, Yarmouk University, Irbid, Jordan; b Department of Pharmaceutics and Pharmaceutical Technology, Faculty of Pharmacy, Yarmouk University, Irbid, Jordan; c Medical Laboratories Department, Prince Hamza Hospital, Amman, Jordan; d Department of Neurology, Al-Bashir Hospital, Amman, Jordan; e Department of Pharmacy Practice, College of Pharmacy, Princess Nourah bint Abdulrahman University, Riyadh, Saudi Arabia.

**Keywords:** Arab, insomnia, people with epilepsy, resilience, risk factors

## Abstract

Although resilience among people with epilepsy (PWE) is important to maintain adequate mental health, however, it has not been unstudied in Jordan. The current study aims to identify the correlates of lower resilience among a cohort of PWE in Jordan, with an emphasis on the demographic variables, clinical variables, and insomnia. This cross-sectional study recruited PWE from Al-Bashir Hospital-Jordan used validated scales for insomnia and resilience. Data from 104 PWE showed that 56 (53.8%) were males, 59 (56.7%) were aged between 18 to 29 years old, and 65 (62.5%) were single. Furthermore, only 9 (8.6%) of the sample received a university education, 60 (57.7%) received a high school education, and 35 (33.6%) received an elementary education. Also, unemployment was evident in 76 (73.1%), and smoking in 37 (35.6%) of the PWE. Also, moderate/severe insomnia was reported in 43 (41.3%), and poor resilience in 54 (51.4%) of the PWE. The multivariable binary logistic regression analysis demonstrated that PWE with lower education showed lower odds (odd ratio = 0.43, 95% CI = 0.22–0.86, *P* = .02) for resilience. Although the current investigation underscored the importance of education in resilience, however, further larger-scale studies have to be pursued to uncover and address other risk factors.

## 1. Introduction

One of the most prevalent neurological disorders in the world is epilepsy. About 50 million people worldwide suffer from epilepsy.^[1]^ Several different medical classes entail seizures, including epilepsy.^[2]^ From benign, self-limiting isolated seizures to those that can be surgically cured or controlled to medically and surgically intractable seizures with or without pervasive developmental disorders, these conditions have a wide range of effects on people.^[3]^ In 2016, there were about 28,226 cases of epilepsy in Jordan.^[4]^ Epilepsy presents serious health concerns in Jordan and worldwide because of its physiological symptoms as well as the related social stigma, the long-term use of antiseizure medications (ASMs), psychological suffering, and quality of life issues.^[5,6]^ Resilience, which is the ability to adapt effectively in the face of difficulty, is essential for people with epilepsy (PWE) to manage the complex difficulties that the condition presents in their day-to-day lives.^[7]^ The significance of resilience as a protective feature that improves well-being in people with chronic illnesses has been highlighted by a recent systematic review.^[8]^ The idea of resilience has become crucial when discussing chronic conditions like epilepsy. It is distinguished by the capacity to tolerate stress, bounce back from failures, and preserve a feeling of direction and optimism.^[9]^ Research has demonstrated that among PWE, resilience is associated with improved psychological outcomes, such as decreased anxiety and sadness.^[10]^

Resilience among PWE is known to be influenced by demographic factors. For example, compared to older adults, younger people frequently exhibit a distinct set of coping mechanisms.^[11]^ Younger patients may be more vulnerable to the social stigmas associated with epilepsy, which can impair their resilience, according to research by Smith et al (2021). On the other hand, older individuals might use life experiences that improve their coping skills.^[12]^ Resilience is significantly impacted by gender disparities as well. According to Lee et al (2021), women with epilepsy may encounter additional obstacles in obtaining care and support, as well as increased societal stigma, which can have a detrimental effect on their resilience.^[13]^ Additionally, socioeconomic status plays a crucial role; lower socioeconomic groups often face higher psychological stress, restricted access to healthcare, and greater health inequities, all of which affect resilience levels.^[14]^

Resilience capacity was shown to be related to insomnia. According to the literature, insomnia is associated with cognitive decline, memory impairment, and high inflammation, all together, these factors could contribute to poor resilience. In addition, the resilience of the families of PWE was found to be affected by insomnia.^[15–17]^

According to our knowledge, no previous studies identified the correlates of resilience level among PWE of Jordan. Therefore, the current study aims to identify the correlates of lower resilience among a cohort of PWE in Jordan, with an emphasis on the demographical variables, clinical variables, and insomnia. By clarifying these connections, this study seeks to improve the quality of life for Jordanian PWE by informing healthcare procedures and resilience-boosting therapies.

## 2. Literature review

Epilepsy is a widespread neurological disorder with various complications that directly affect the quality of life. On one side, the frequency and the severity of seizures determined the quality of life in many patients, that is, PWE with controlled seizures reported better quality of life compared to their peers with uncontrolled seizures.^[18]^ On the other hand, other research did not relate quality of life to seizure control, but rather with resilience skills demonstrated by PWE.^[19]^ For example, literature indicated that variables in Kumpfer resilience framework model significantly determined the life satisfaction of PWE.^[7]^ Resilience could be simply defined as the ability to “get back” on track after adversity is gaining attention concerning long-term medical conditions such as chronic pain, diabetes, and cancer.^[20–22]^

According to a four-year follow-up study, the presence of depression and medication adverse effects predicted poor resilience in PWE. Therefore, addressing the modifiable risk factors for poor resilience in PWE warrants to a great extent to resilience skills and subsequently the quality of life.^[23]^

In this context, self-compassion is believed to reduce the risk of depression and increase the odds of higher resilience. A cross-sectional investigation comprising 270 PWE concluded that self-compassion predicted lower odds ratios for anxiety and depression on one side and superior resilience in PWE.^[24]^ According to the literature, self-compassion strategies provide psychological adaptation by accepting disease or adversity as something universal experienced by thousands of PWE worldwide. This is believed to mitigate negative reactions, minimize discomfort, and self-criticism. Simultaneously, this will increase optimism and self-efficacy.^[25–28]^

In addition, a study by Sinead Pembroke identified strategies for resilience in PWE, especially in the early diagnosis period. Processing the emotional responses to the epilepsy diagnosis was prominent, as finding out more information about the disease relieved the patients. Being diagnosed helped them understand their symptoms. Although this did not cancel out the feelings of sadness, their sadness and anger were accompanied by relief that helped them adjust their lifestyle. Also, according to Sinead Pembroke et al, PWE adopted strategies to become comfortable with their epilepsy, which confirms that resilience is an interactive process rather than a personal trait. These strategies included understanding their seizure type, perceiving their epilepsy as ordinary, and talking about their epilepsy at ease with others.^[29]^ Therefore, the investigation of the factors associated with resilience is important to enhance resilience capacity and positively contribute to the well-being of PWE.

## 3. Methods

### 3.1. Study settings and design

The recruitment of PWE was carried out from Al-Bashir Hospitals Clinics-Amman-Jordan. The study protocol gained approval from the Institutional Review Board of Yarmouk University (no. 692). All methods were performed following the Declaration of Helsinki. Data were collected between April and July 2024. The researchers approached the PWE to explain the objective of the study. Next, the interested filled out the online study instrument using Google Forms. The study sample size was informed by previous studies,^[5,30,31]^ also, the sample size was calculated using G-power. Based on the using of multiple regression, effect size of 0.15, power of 95, and significant level of 0.05; the G-power indicated the need for at least 89 participants.

### 3.2. Inclusion criteria

PWE diagnosed as per the American Academy of Neurology guidelines with Generalized Tonic-Clonic seizures for at least 6 months, and who are attending the neurology outpatient clinics at AL-Bashir Hospitals were included in the study.

### 3.3. Study instrument

The study instrument was composed of the following 4 sections as described below:

- Demographical information: The participants were asked about their sex, age, marital status, highest education received, current employment status, and smoking status.
- Clinical information: After applying the inclusion criteria as mentioned above, PWE were asked to provide information about the duration of the disease since diagnosis and the name of the currently used ASM.
- Insomnia: The insomnia severity was assessed using the translated, validated, and reliable Arabic version of the Insomnia Severity Index. Respondents demonstrating a score of 15 and higher for the 7 questions were classified as having moderate/severe insomnia.^[6,32]^
- Resilience: This is the outcome variable (dependent variable) of the study. The resilience level was evaluated using the Arabic version of the Connor–Davidson Resilience Scale, consisting of 25 statements rated on a Likert scale ranging from 0 (“totally incorrect”) to 5 (“always correct”). Any score of more than 50 is considered resilient.^[33,34]^

## 4. Data analysis

The demographical and clinical data of the PWE were categorical variables and therefore were described as frequencies and percentages. The associations between the independent variables of the PWE and the dependent variable (resilience) were examined through the χ^2^ test. Next, the variables showing potential confounding were inserted into the multivariable binary logistic regression analysis that finally consisted of only the variables showing statistical significance of *P* < .05. The confidence intervals (CIs) were set at 95% and the significance at *P* < .05. Data were analyzed using Statistical Package for the Social Sciences version 21.

## 5. Results

### 5.1. Study sample characteristics

Initially, a total of 181 PWE were approached, 77 did not meet the inclusion criteria, therefore, the analysis of data was conducted from 104 PWE who showed the following: 56 (53.8%) were males, 59 (56.7%) were aged between 18 to 29 years old, and 65 (62.5%) were single. Furthermore, only 9 (8.6%) of the sample received university education, 60 (57.7%) received high school education, and 35 (33.6%) received elementary education. Also, unemployment was evident in 76 (73.1%), and smoking in 37 (35.6%) of the PWE. Also, moderate/severe insomnia was reported in 43 (41.3%), and poor resilience in 54 (51.4%) of the PWE. Detailed information is found in Table 1.

**Table 1:** The associations between the patients’ variables (n = 104), distributed across the dependent variable (resilience level) using χ^2^ test.

Factor	Category	Lower resilience (n = 54, 51.4%)	Higher resilience (n = 51, 48.6%)	χ^2^	*P*-value
Gender	Male	31 (57.4)	25 (50)	0.57	.29
	Female	23 (42.6)	25 (50)		
Age	18–29 yr	34 (63)	25 (50)	1.78	.24
	≥30 yr	20 (37)	25 (50)		
Marital status	Single	37 (68.5)	28 (56)	1.73	.23
	Married	17 (31.5)	22 (44)		
Highest education achieved	University	4 (7.4)	5 (10)	8.06	.02
	High school	25 (46.3)	35 (70)		
	Elementary school	25 (46.3)	10 (20)		
Employment	Employed	12 (22.2)	16 (32)	1.26	.18
	Unemployed	42 (77.8)	34 (68)		
Smoking status	Nonsmoker	34 (63)	33 (66)	0.10	.83
	Smoker	20 (37)	17 (34)		
Disease diagnosed	<5 yr	19 (35.2)	20 (40)	0.26	.68
	≥5 yr	35 (64.8)	30 (60)		
Moderate/severe insomnia?	No	32 (59.3)	30 (58.8)	0.002	1
	Yes	22 (40.7)	21 (41.2)		
Using sleep aid?	No	47 (87)	45 (90)	0.22	.76
	Yes	7 (13)	5 (10)		
Carbamazepine?	No	36 (66.7)	33 (66)	0.005	1
	Yes	18 (33.3)	17 (34)		
Lamotrigine?	No	41 (75.9)	39 (78)	0.06	.82
	Yes	13 (24.1)	11 (22)		
Levetiracetam?	No	33 (61.1)	26 (52.0)	0.88	.43
	Yes	21 (38.9)	24 (48.0)		
Valproate?	No	38 (70.4)	43 (86)	3.68	.06
	Yes	16 (29.6)	7 (14)		
Topiramate?	No	49 (90.7)	45 (90)	0.01	1
	Yes	5 (9.3)	5 (10)		

N = number.

### 5.2. Correlates of poor resilience

The identification of the correlates associated with poor resilience; a preparatory association was performed using chi-square analysis as in Table 1. This analysis revealed that “lower education level” and “valproate” were associated with resilience level as they showed a *P*-value below the threshold of .1. Next, these independent variables were used to build a multivariable binary logistic regression model. The final model contained “lower education” as the only independent variable significantly (odd ratio = 0.43, 95% CI = 0.22–0.86, *P* = .02) associated with resilience. In other words, lower education years were negatively associated with high resilience levels in the PWE examined. Refer to Table 2.

**Table 2:** The final model of the multivariable regression model showing the association between “education” as an independent variable and the resilience level (dependent variable).

Independent variable	*B*	Wald	OR	95% CI	*P*
Lower education	−0.84	5.72	0.43	0.22–0.86	.02
Constant	0.97	4.06	2.64	–	.04

CI = confidence interval, OR = odd ratio.

## 6. Discussion

This study identified predictive factors of resilience in PWE in Jordan and underlined the demographic data and the possibility of ASM involvement. The results showed a significant relationship between lower education levels and higher resilience in PWE. This finding confirms that prior research has focused on education as a determining variable in the improvement of resilience and quality of life among people with chronic diseases. The higher the level of education, the more empowered an individual is to handle health challenges in terms of both ability and knowledge. In the context of epilepsy, higher education levels have been associated with better self-management strategies and coping mechanisms, which are important for building resilience. On the contrary, at a low level of education, there may be reduced exposure to vital information and resources, which increases the psychosocial burden of epilepsy. This supports the evidence from numerous studies that education plays a protective role in fostering resilience among PWEs.

Therefore, these findings need to be translated into practice by incorporating comprehensive educational interventions into care plans regarding epilepsy.

Individualized educational programs that address the needs of children, adults, and caregivers could enhance resilience. Addressing basic knowledge of the disease, seizure types, potential triggers of seizures, treatment adherence, and educational programs are necessary. Systematic reviews have shown that organized educational interventions can improve epilepsy knowledge in patients and their families, leading to improvements in patient care.

Such a blend in the delivery method can be made available in face-to-face workshops, online webinars, print brochures, and mobile app interactive displays. There is some supportive evidence that the combination of traditional teaching and technology-based learning results in better engagement and retention. This will be the case, especially for individuals with lower educational backgrounds who may need easily accessible education tools to bridge the knowledge gap and thereby be better equipped for good self-management of their condition. The involvement of caregivers and family members is also important. Caregiver-oriented educational interventions may help improve knowledge regarding the management of epilepsy, thus enabling them to provide better assistance in emergencies or daily routines of care. This wider circle of support can positively influence the resilience of PWE because patients feel more secure in such an understanding environment. Various studies have documented that focused training on cognitive development for caregivers translates into increased confidence and competence relevant to the resilience of children in care.

Another successful approach is collaboration between schools and educational institutions. They allow epilepsy education to be incorporated into school health programs; thus, teachers, staff members, and students become informed about seizure management and first-aid approaches. This provides great support to students with epilepsy.

Such a proactive approach minimizes stigma, increases social acceptance, and is important for the psychological well-being of younger PWE. Further development of seizure action plans for individual students will reinforce this effort and instill a sense of preparedness in school personnel.

Follow-up visits and support groups, such as peer support groups, may help sustain the positive effects of initial educational programs. Ongoing education may fix knowledge and adapt to alterations in patient conditions or changes in treatment plans, resulting in long-term resilience that is finding a boost. Such follow-up consultation also allows for discussions on new challenges that have emerged and sharing of experiences, which is a way of building community and one aspect of collective problem solving in both patients and caregivers^[35]^

This would include the use of health care-provided pre- and post-intervention assessments to determine the improvement in knowledge and practical management skills through educational interventions. These come in the form of quizzes or surveys meant to assess the aforementioned knowledge and management skills improvements. Assessments could be a feedback mechanism for future educational programs such that they remain appropriate according to needs. There is support from studies that such an assessment has helped modify both the content and method of delivery of education with subsequent long-term improvements in PWE. It will also be possible to increase public awareness of epilepsy and dispel myths, thereby decreasing stigma through community outreach programs. Such initiatives make PWE feel supported and accepted by their community. This is important for the psychological resilience of epilepsy patients. Community events, workshops, and public health campaigns conducted for the education of the greater community may create a snowball effect in society, increasing understanding and acceptance.^[36]^

Although the findings from this study may point toward the significance of education in promoting resilience among PWEs, further consideration was taken regarding the use of ASM. Initial analyses showed no statistically significant association between the use of valproate and the level of resilience in the multivariable regression model. All these findings disagree with those of studies that claimed the cognitive adverse effects of valproate, which would supposedly have depressive effects on resilience.^[37]^

This may indicate that although the choice of medication is very important concerning seizure control, it is not as important when considering psychological well-being and strength as other factors such as educational background and support systems.^[38]^ The study indicated that resilience is significantly influenced by the level of education among PWE in Jordan. The use of ASM is an important part of seizure management, but more decomposition using current data needs to be performed to form the role of ASM in resilience. Healthcare strategies, including comprehensive education through the involvement of the caregiver and psychological approaches to epilepsy treatment, would provide more holistic support for PWE, leading to greater resilience and quality of life.

Several key elements should be incorporated to ensure that the discussion effectively covers educational interventions for caregivers of pediatric epilepsy patients. To begin with, there is a need to have some supporting statistical data in the form of p-values or effect sizes that support the claims of improvement in knowledge and resilience among caregivers. For example, one published article^[39]^ highlighted how the implementation of organized educational programs leads to a significant enhancement of knowledge on epilepsy among caregivers, with an effect size of 1.23, *P* < .001. This may be very helpful in setting any findings against the backdrop of global trends in epilepsy management to further assess the generalizability of such findings across various cultural contexts. A comparative study would, therefore, be indicative of the fact that there are requirements and outcomes in the education of these populations.^[40,41]^ There is also a need to extensively study how ASM and educational intervention interface. Initial analyses did not find an association between valproate and resilience, but there is a need for further investigation as to whether medication adherence plays a role in varying educational outcomes, has been reported^[42,43]^ Furthermore, the addition of psychological support to educational strategies may provide holistic resilience, study^[41]^ noted that combined interventions substantially improved knowledge and emotional well-being in caregivers. Long-term assessments of educational interventions are relevant to reveal their maintenance and effectiveness over a period of time or period.^[36,37]^ Problematic issues in the implementation cycle, such as resource constraints and cultural attitudes toward epilepsy, must also be taken into consideration to provide a more realistic framework for the effective attainment of educational outcomes. Lastly, through the institution of robust feedback mechanisms, continuous refinement of the educational programs based on the experiences of caregivers would ensure appropriateness and effectiveness. This would thereby help present a more holistic perspective in the discussion of ways in which the resilience of pediatric epilepsy patients could be enhanced through appropriate caregiver education.

## 7. Conclusion

Although the current study brought novel insights, however, it has some limitations. For example, one limitation of this study is its cross-sectional design, which only allows for the establishment of the relationship between 2 or more variables without establishing causality, rather than cause and effect. A follow-up design could provide more insights about the dynamics between covariates and resilience. Also, the sample was region-specific; hence, therefore, generalization of the results may be limited. Furthermore, the resilience level was assessed using a self-completed scale, which could lead to a possible bias or exaggerated results. However, any potential bias was minimized by solid statistical analysis and validated scales. Further studies with a longitudinal design using larger and more diverse populations are necessary to validate the reported findings and investigate other variables that may influence resilience in PWE. Future perspectives include integrating mental health care to screen, diagnose, and follow up PWE during their journey. In addition, the continuous education of PWE and their families and friends provide additional support for this fragile population.

In conclusion, the study of resilience in PWE of Jordan is still in its infancy, although the current investigation underscored the importance of education in resilience, however, further larger-scale studies have to be pursued to uncover and address other risk factors.

## Acknowledgments

The current work was supported by Princess Nourah bint Abdulrahman University Researchers Supporting Project number (PNURSP2025R485), Princess Nourah bint Abdulrahman University, Riyadh, Saudi Arabia.

## Author contributions

**Conceptualization:** Omar Gammoh, Alaa A. A. Al-Jabali, Sireen Abdul-Rahim Shilbayeh.

**Data curation:** Hayam A. AlRasheed.

**Formal analysis:** Mariam Al-Ameri, Nada A. AlSaleh, Alaa A. AlSharif.

**Funding acquisition:** Omar Gammoh, Hayam A. AlRasheed, Sireen Abdul-Rahim Shilbayeh.

**Investigation:** Alaa A. A. Al-Jabali, Hanan Abu-Alshaikh, Wail Ennab, Nada A. AlSaleh.

**Methodology:** Mariam Al-Ameri, Alaa A. AlSharif, Hayam A. AlRasheed.

**Project administration:** Sireen Abdul-Rahim Shilbayeh.

**Resources:** Wail Ennab.

**Software:** Wail Ennab.

**Supervision:** Omar Gammoh, Sireen Abdul-Rahim Shilbayeh.

**Validation:** Mariam Al-Ameri, Hanan Abu-Alshaikh, Wail Ennab.

**Visualization:** Alaa A. A. Al-Jabali.

**Writing – original draft:** Omar Gammoh, Mariam Al-Ameri, Alaa A. A. Al-Jabali, Hanan Abu-Alshaikh, Wail Ennab, Nada A. AlSaleh, Alaa A. AlSharif, Hayam A. AlRasheed, Sireen Abdul-Rahim Shilbayeh.

**Writing – review & editing:** Omar Gammoh, Mariam Al-Ameri, Alaa A. A. Al-Jabali, Hanan Abu-Alshaikh, Wail Ennab, Nada A. AlSaleh, Alaa A. AlSharif, Hayam A. AlRasheed, Sireen Abdul-Rahim Shilbayeh.
